# Novel therapeutic modulators of astrocytes for hydrocephalus

**DOI:** 10.3389/fnmol.2022.932955

**Published:** 2022-09-26

**Authors:** Yijian Yang, Chuansen Wang, Rui Chen, Yuchang Wang, Changwu Tan, Jingping Liu, Qinghua Zhang, Gelei Xiao

**Affiliations:** ^1^Department of Neurosurgery, Affiliated Nanhua Hospital, Hengyang Medical School, University of South China, Hengyang, China; ^2^Department of Neurosurgery, Xiangya Hospital, Central South University, Changsha, China; ^3^Diagnosis and Treatment Center for Hydrocephalus, Xiangya Hospital, Central South University, Changsha, China; ^4^National Clinical Research Center for Geriatric Disorders, Xiangya Hospital, Central South University, Changsha, China; ^5^Department of Neurosurgery, Huazhong University of Science and Technology Union Shenzhen Hospital, Shenzhen, China; ^6^The 6th Affiliated Hospital of Shenzhen University Health Science Center, Shenzhen, China

**Keywords:** astrocytes, hydrocephalus, cerebrospinal fluid, pathogenesis, neuroinflammation

## Abstract

Hydrocephalus is mainly characterized by excessive production or impaired absorption of cerebrospinal fluid that causes ventricular dilation and intracranial hypertension. Astrocytes are the key response cells to inflammation in the central nervous system. In hydrocephalus, astrocytes are activated and show dual characteristics depending on the period of development of the disease. They can suppress the disease in the early stage and may aggravate it in the late stage. More evidence suggests that therapeutics targeting astrocytes may be promising for hydrocephalus. In this review, based on previous studies, we summarize different forms of hydrocephalus-induced astrocyte reactivity and the corresponding function of these responses in hydrocephalus. We also discuss the therapeutic effects of astrocyte regulation on hydrocephalus in experimental studies.

## Highlights

– Astrocytes are understudied in hydrocephalus but plays an essential role in the condition.
– Their specific pathologic contributions and targeting astrocytes as a therapy for hydrocephalus are summarized for the first time.

## Introduction

Hydrocephalus is a cerebrospinal fluid (CSF) functional disorder that causes ventricular dilation and intracranial hypertension (sometimes, it may not be associated with it). Hydrocephalus is commonly caused by craniocerebral trauma, intracranial space-occupying lesions, or intracranial infection. Periventricular gliosis, including astrocytes, microglia hypertrophy, and hyperplasia, has been reported in human and animal models of hydrocephalus (Del Bigio, 2001; Deren et al., 2010; Eskandari et al., 2011; Zhan et al., 2020); this suggests that gliosis is a significant neuropathological feature of hydrocephalus. Changes in glial cells, especially astrocytes, may play an essential role in the pathogenesis of hydrocephalus.

Astrocytes are the most widely distributed type of cells in the mammalian brain and the most numerous type of glial cells (Zhou et al., 2019). They play an important role in various physiological activities, such as maintaining ion homeostasis and participating in cerebrospinal fluid circulation (Volterra et al., 2014; Plog and Nedergaard, 2018; Lafrenaye and Simard, 2019). They can regulate their own metabolic activities and control the synthesis and reuptake of neurotransmitters and neurotrophic factors through the transduction of various receptors and signaling pathways (Pekny and Pekna, 2014; Li-Na et al., 2017; Durkee and Araque, 2019). Moreover, their ability to regulate inflammatory cytokines and free radical release play an important role in the pathological process of central nervous system (CNS) diseases (Sofroniew, 2015; González-Reyes et al., 2017; Cabezas et al., 2019). Research showed extensive gliosis in communicating hydrocephalus, which is related to the pro-inflammatory role of astrocytes (Xu et al., 2012b; Xu et al., 2015). Reactive astrocyte proliferation is a repair and healing response to brain tissue injury, mainly manifested as fibrous astrocyte proliferation, which eventually becomes a glial scar with strong positive staining for glial fibrillary acidic protein (GFAP). Moreover, the CNS responds to different injury situations by causing different changes in astrocytes, suggesting that astrocytes are important response cells in CNS injury. Therefore, targeting astrocytes for the treatment of hydrocephalus may show some promise.

In this manuscript, based on prior experimental studies, we first described the morphological characteristics of astrocytes under physiological conditions and their changes in hydrocephalus. We then focused on the astrocyte responses to hydrocephalus and the consecutive functions of these responses in hydrocephalus development. They finally briefly discussed the therapeutic effects of regulating astrocytes on hydrocephalus in animal model studies.

## Astrocytes in the central nervous system

Astrocytes are distributed throughout the CNS and are involved in structural support, blood–brain barrier (BBB) formation, extracellular environment maintenance, anti-oxidative stress, and many other activities (Pekny and Nilsson, 2005). Astrocytes give off many long, branching processes from the cell body, which extend and fill the space between the cell body and its processes. Astrocytes can be divided into two types: fibrous astrocytes and protoplasmic astrocytes (Borggrewe et al., 2021). Fibrous astrocytes are mostly distributed in the cortex of the spinal cord with elongated protrusions and few branches. Protoplasmic astrocytes are mainly distributed in gray matter, with stubby cell projections and many branches (Miller and Raff, 1984).

The ends of the protuberance are often enlarged to form the end feet and attach to the adjacent capillary wall or the inferior membrane of the ependyma. Three-dimensional electron microscopy reconstruction of the endings of vessels in the rat hippocampus revealed that the end feet interdigitated without leaving any slits between them (Mathiisen et al., 2010). Astrocytes have extensive gap junctions composed mainly of connexins (CXs). These gap junctions are enriched in the endfeet of astrocytes, which enwrap the blood vessels’ walls and provide a perivascular route (Liu and Chopp, 2016). Small molecules can pass through gap junctions and participate in cell-to-cell communication.

The mitochondria in endfeet differ markedly in size and shape. The 3D reconstructions found two main types of mitochondria: small/ovoid and elongated (Mathiisen et al., 2010). Others show very complex shapes. Different mitochondria intertwine and form large bundles that fit tightly into the endfoot membranes around the blood vessels.

Astrocytes are the key response cells in CNS injuries since they respond to inflammatory stimuli by releasing pro-inflammatory molecules. Therefore, they are greatly involved in the development of hydrocephalus. They are associated with neuroinflammation by producing various pro-inflammatory molecules (Cekanaviciute et al., 2014b; Pekny and Pekna, 2014). They are also involved in fluid regulation because over-expressing aquaporin 4 (AQP4) can regulate cell swelling or reduce volume (Petzold et al., 2006). Besides, they can provide antioxidant protection by secreting neurotrophic factors and antioxidants (Sofroniew, 2015).

The above briefly describes the main functions of astrocytes, which are closely related to the pathogenesis of hydrocephalus and are the focus of our research and will be described in detail below.

## Abnormal astrocytes in hydrocephalus

**FIGURE 1:**
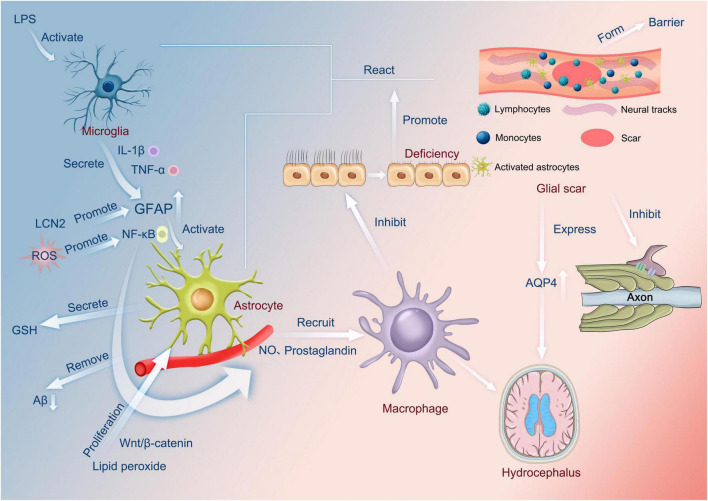
Functions of astrocytes. LPS activates microglia to secrete cytokines, such as IL-1β and TNF-α, which increase the amount of GFAP. In addition, LCN2 also promotes the increase in GFAP. The increased GFAP activates astrocytes *via* NF-κB, which, in turn, recruits macrophages through the release of nitric oxide or prostaglandins. Lipid peroxide can also affect macrophages *via* Wnt/β-catenin in this process, and reactive oxygen species can also influence the production of nitric oxide and prostaglandin by promoting NF-κB and then affecting macrophages. Macrophages can inhibit normal cilia, and cilia abnormalities can occur, encouraging the interaction of microglia and astrocytes and the appearance of glial scars. On the one hand, this can produce a barrier. On the other hand, it can inhibit axonal neurotransmission and, most importantly, contribute to the overexpression of AQP4, causing hydrocephalus. In addition, astrocytes have the function of secreting glutathione and scavenging amyloid. LPS, Lipopolysaccharide; lL-1β, Interleukin-1β; TNF-α, Tumor necrosis factor-α; GFAP, glial fibrillary acidic protein; LCN2, Lipoprotein 2; ROS, Reactive oxygen species; GSH, glutathione; Aβ, amyloid protein; NO, nitric oxide; AQP4, aquaporin 4.

In hydrocephalus, factors such as hypoxia promote the activation of astrocytes. In patients with hydrocephalus, the astrocytes show significant edema and phagocytic activity (Castejon, 1994; Castejón, 2010). Furthermore, active astrocytes express more intermediate filament proteins, including glial fibrillary acidic protein (GFAP), vimentin, nestin, and many other altered molecules (Petzold et al., 2006; Liu and Chopp, 2016; Eide and Hansson, 2018). Reactive astrocytes were mainly found in periventricular white matter and cortical gray matter (Del Bigio, 2010). Activated astrocytes can recruit other astrocytes to migrate to the site, forming a glial scar (Pekny and Nilsson, 2005). The number of reactive astrocytes may be reduced after shunt, but they cannot return to normal conditions for a long time (Eskandari et al., 2011). Therefore, surgical treatment cannot completely reverse the reaction even in the long term (Figure 1).

Interestingly, neuroinflammation-induced reactive astrocytes exhibit the property of secreting neurotoxins that promote the death of neurons and oligodendrocytes (Liddelow et al., 2017). In contrast, ischemia-induced reactive astrocytes increased neurotrophic factor expression and exhibited more neuroprotective properties (Zamanian et al., 2012).

The functional role of the glial scar is also controversial. On the one hand, it can secrete molecules such as chondroitin sulfate proteoglycans that inhibit axon regeneration (McKeon et al., 1991). On the other hand, it acts as a barrier to prevent the spread of inflammatory cells and factors into healthy tissue (Burda et al., 2016). Loss of proliferating astrocytes leads to significantly increased levels of amyloid-β, indicating that reactive astrocytes are involved in the clearance of amyloid peptides (Katsouri et al., 2020).

Endoependymal exfoliation in hyh mutant mice caused adjacent astrocytic proliferation. These astrocytes expressed specific glial markers and formed a layer of surface cells to replace the lost ependyma (Roales-Buján et al., 2012). Reactive astrocytes on the removed ependymal surface showed a small but significant increase in AQP4 compared with the ependymal of wild-type mice (Roales-Buján et al., 2012). This may be an adaptive change in response to ependymal damage. Whether this change is due to the direct effect of ependymal damage or the mechanical compression of hydrocephalus needs to be studied.

Electron microscopy was used to analyze cerebral cortex slices from 30 idiopathic normal pressure hydrocephalus (iNPH) patients. The results showed that the number of normal mitochondria decreased significantly in the astrocytic endfeet of iNPH patients compared to normal individuals, accompanied by a significant increase in the number of pathological mitochondria (Hasan-Olive et al., 2019a). These changes were obviously related to the degree of astrogliosis. This indicates an energy metabolism disorder in the astrocytes of patients with iNPH (Wang et al., 2020). Also, pathological mitochondria were significantly and negatively correlated with the perivascular expression of AQP4 and dystrophin-71 (Hasan-Olive et al., 2019a).

## Astrocytes regulate neuroinflammation in hydrocephalus

Many neurological diseases, including hydrocephalus, are associated with neuroinflammation, but the exact mechanisms are not fully understood. In the CNS, astrocytes play a major role in inflammation (Sun et al., 2021). In animal models, significant inflammatory responses have been demonstrated in all stages of hydrocephalus, in which reactive astrocytes may play a central role (Lopes Lda et al., 2009; Deren et al., 2010; Olopade et al., 2012). After intraperitoneal injection of lipopolysaccharide (LPS), the expression of pro-inflammatory cytokines in microglia was found to peak 2–4 h after injection, but the peak of pro-inflammatory cytokine expression in astrocytes occurred 12–24 h after injection (Norden et al., 2016). This might indicate that microglia are involved in activating astrocytes by secreting pro-inflammatory factors in the inflammatory response of the CNS. Astrocytes can be activated by various pro-inflammatory mediators, such as IL-1β (John et al., 2004). Microglia are the main source of IL-1β, mainly expressed in astrocytes and perivascular macrophages 4 weeks after hydrocephalus induction (Olopade et al., 2019). At the same time, astrocytes can produce a variety of pro-inflammatory molecules, such as prostaglandins and nitric oxide (NO), to amplify neuroinflammation (Sofroniew, 2015; Michinaga and Koyama, 2019).

Astrocyte signaling pathways appear to be regulated by common downstream transcriptional regulators during inflammation (Linnerbauer et al., 2020). Nuclear factor-κB (NF-κB) is greatly involved in the response of astrocytes to inflammatory stimuli and other injuries. NF-κB is a major regulator of cell survival, differentiation, and proliferation, as well as innate and adaptive immunity. The NF-κB signal of astrocytes can be directly activated by various pro-inflammatory factors such as TNF-α, IL-1β, and TLR signals (Kawai and Akira, 2007; Shih et al., 2015). Activation of NF-κB in astrocytes induces the expression of pro-inflammatory mediators, leading to the recruitment of macrophages, thereby inhibiting ependymal cilia formation and, ultimately, hydrocephalus formation (Lattke et al., 2012). However, in this study, activation of NF-κB caused hydrocephalus only in the developing brain and did not show significant lateral ventricular dilation in mature rats. However, NF-κB may promote hydrocephalus through mechanisms other than inhibiting ependymal cilia formation, such as promoting the secretion of other pro-inflammatory factors.

A study has shown that neuroinflammation is found in ventricular dilation in rats with hydrocephalus and suggests that it is involved in the upregulation of IL-1β secreted by astrocytes in the early stages of the disease (Olopade et al., 2019). In this study, IL-1β was significantly increased at weeks 1 and 4, followed by downregulation at week eight, which seems to be consistent with the clinical characteristics of posthemorrhagic hydrocephalus in premature infants (Schmitz et al., 2007). It suggests that neuroinflammation in the later stage of hydrocephalus is relieved. In some disease models, astrocytes secrete transforming growth factor-β to reduce disease-associated inflammatory responses (Cekanaviciute et al., 2014a,b). However, transforming growth factor-β is closely related to subarachnoid fibrosis in the development of hydrocephalus (Cherian et al., 2004; Zhan et al., 2020; Wang et al., 2021). In subarachnoid hemorrhage, the body responds to the injury by releasing various factors through many different pathways to activate astrocytes, which, in turn, repair the BBB.

Lipoprotein 2 (LCN2) is an iron-carrier binding protein that plays a role in endogenous iron chelation. It is an acute-phase protein expressed by astrocytes after ischemic stroke, cerebral hemorrhage, and neuroinflammation (Dong et al., 2013; Ni et al., 2015). It is reported that LCN2 knock-out mice injected with hemoglobin showed less ventricular dilation and fewer activated astrocytes and amoeba microglia compared with control mice (Shishido et al., 2016). Another recent study has demonstrated that LCN2 deficiency reduces neuroinflammation by reducing glial and microglial cell activation in a model of systemic inflammation (Jin et al., 2014). Previous studies have also shown that LCN2 increases glial fibrillary acid protein (GFAP) expression and promotes activation of astrocytes and microglia (Xing et al., 2014).

## Astrocytes regulate abnormal expression of aquaporins in hydrocephalus

Aquaporins (AQPs) are non-selective bidirectional channel proteins that allow water to diffuse passively and thus allow net fluxes of water driven by concentration gradients. There are three main types of AQPs in the CNS: AQP1, AQP4, and AQP9 (Potokar et al., 2016). AQP4 is mainly located in the endfeet of astrocytes, with a severalfold higher density of the membrane domains facing capillaries than membranes facing the neuropil (Nielsen et al., 1997). This polarization depends on the α-syntrophin, an intracellular component of the dystroglycan complex (Neely et al., 2001). This spatial distribution may be beneficial in improving the efficiency of CSF-interstitial fluid exchange. Astrocytes overexpressing AQP4 have a greater ability to regulate cell swelling or reduce volume (Lisjak et al., 2017). Also, basal brain water content was increased in mice with a complete loss of AQP4 water channels (Vindedal et al., 2016). These suggest that AQP4 in astrocytes may be involved in fluid regulation in the brain. AQP4 may also be involved in astrocyte migration. The leading edge of AQP4 expression was increased in migrating astrocytes, and the ability of AQP4-null migration was significantly reduced compared to wild-type astrocytes (Saadoun et al., 2005). Inhibition of glial scarring was also observed in AQP4-null mice, which may be related to the inhibition of astrocyte migration by AQP4 reduction (Auguste et al., 2007).

Moreover, AQP4 may be involved in the regulation of inflammation. Astrocyte cultures from wild-type mice released more tumor necrosis factor-α (TNF-α) and interleukin-6 (IL-6) than those from AQP4-null mice. In animal models, lipopolysaccharide (LPS)-treated AQP4-inactivated mice also showed a smaller inflammatory response (Li et al., 2011). It is also reported that deletion of AQP4 is associated with a distinct inflammatory response of the retina (Pannicke et al., 2010). Thus, AQP4 may play different roles in the regulation of inflammation under different pathological conditions.

The expression of AQP4 by the endfeet of astrocytes changes dynamically in hydrocephalus. In kaolin-induced hydrocephalus rats, the abundance of AQP4 in the periventricular area and cortex was significantly decreased on day two after treatment with kaolin on day one but increased significantly after week 2 (Skjolding et al., 2010). On the other hand, Mao et al. investigated the effect of obstructive hydrocephalus on the expression of AQP4 in rats and found that the mRNA levels of the AQP4 channel were changed (Mao et al., 2006). But surprisingly, this was not accompanied by an increase in protein levels. The most likely explanation is that a major redistribution of AQP4 occurs in hydrocephalus rather than an increase in overall abundance. This redistribution may be a protective mechanism against the accumulation of CSF.

Normally, AQP4 is expressed primarily in the terminal foot of astrocytes. However, in the case of hydrocephalus, this polarization may change. Immunogold cytochemical analysis of AQP4 in cortical brain biopsies from 30 iNPH patients and 12 reference individuals showed that AQP4 density was reduced in astrocytic endfoot membranes along cortical microvessels of the iNPH brain compared to the control group (Hasan-Olive et al., 2019b). As β-dystroglycan-immunopositivity in brain vessels coincides with the reactive glial reaction; this depolarization may be due to the activation of astrocytes (Szabó and Kálmán, 2008; Kálmán et al., 2011). This may indicate an obstruction of perivascular CSF-interstitial fluid circulation.

Interestingly, the expression profile of AQP4 in rat brain tissue seems to differ from that in human tissue. In human hydrocephalus samples, AQP4 fluorescence signals were present throughout the astrocyte membrane. In rats with hydrocephalus, the fluorescence signal of AQP4 was strongly polarized to the perivascular foot of astrocytes (Skjolding et al., 2013). One possible explanation is that this may be due to diseases having different characteristics in different species. Therefore, AQP4 depolarization occurs in the mouse model of iNPH (Kress et al., 2014). Moreover, because progressive AQP4 depolarization occurs throughout the physiological aging process of mice, further age-matched human studies are needed to determine whether the AQP4 pathological depolarization is a characteristic response of iNPH rather than a feature of aging (Kress et al., 2014).

Overexpression of AQP4 in CSF may also be present in patients with congenital hydrocephalus. CSF samples were collected from the lateral ventricles of 13 full-term t infants. Western-blot analysis showed that AQP4 expression was higher in traffic hydrocephalus than in the control group but was not significant in obstructive hydrocephalus (Castañeyra-Ruiz et al., 2013). This AQP4 movement may be a consequence of ependyma denudation. Loss of communication between ependymal cells leads to ependymal dissection and entry of AQP4 into the CSF. The ependymal deletion was accompanied by microglia and astrocyte reactions. Subependymal astrocytes proliferate to form a glial scar covering the ventricle surface, and the replacement of the ependymal reduces the chance of AQP4 entering CSF (Páez et al., 2007; Roales-Buján et al., 2012).

## Astrocytes regulate oxidative stress in hydrocephalus

As the brain consumes more energy than any other organ in the body, it produces large amounts of free radicals, such as reactive oxygen species (ROS) or reactive nitrogen. However, oxidative damage can occur when the production of free radicals outstrips the brain’s ability to clear them. Although the role of oxidative stress in hydrocephalus has not been clearly understood, more studies suggest that oxidative stress may be one of the causes of hydrocephalus (Socci et al., 1999; Li et al., 2014; Guzelcicek et al., 2020). Oxidative stress produces large amounts of ROS and lipid peroxidation products, which may cause great damage to proteins, lipids, and DNA. ROS have also been found to be involved in crosstalk with NF-κB signaling, which links neuroinflammation to oxidative stress (Morgan and Liu, 2011). Lipid peroxidation products may also induce reactive astrocyte proliferation in hydrocephalus by activating the Wnt/β-catenin pathway (Xu et al., 2015; Suryaningtyas et al., 2020). Wnt/β-catenin signaling also plays an important anti-inflammatory and pro-inflammatory role. The regulation of the NF-κB pathway may be involved in this effect (Ma and Hottiger, 2016).

There is also evidence that the overproduction of NO may be involved in the pathological process of hydrocephalus (Del Bigio et al., 2012). In the CNS, NO is mainly produced by neuronal NO synthase, inducible NO synthase produced by activated microglia, and endothelial NO synthase (Barnham et al., 2004). Although NO can alleviate hypoxia by dilating blood vessels, NO may also be oxidized into peroxynitrite ONOO, causing serious damage to cells. Furthermore, the increase of ROS during neuroinflammation may lead to the activation of NF-κB, which, in turn, induces the overexpression of NO synthase in astrocytes and microglia, especially inducible NO synthase, leading to the production of superoxide (González-Reyes et al., 2017).

A sustained increase in ventricular volume was observed in rat pups reared under chronic sublethal hypoxia (Ment et al., 1998). Nerve cell-specific hypoxia-inducible factor-1α deficient mice showed severe hydrocephalus with memory loss (Tomita et al., 2003), suggesting that hypoxia may contribute to the development of hydrocephalus. Cortical compression caused by ventricular enlargement may cause local tissue ischemia and hypoxia, producing free radicals. The detection of hypoxia and free radical production markers in hydrocephalus rats also suggests that hypoxia mechanisms play a role in hydrocephalus brain injury (Del Bigio et al., 2012). However, no upregulation of antioxidant enzymes was detected in this model. The protective effect of antioxidant enzymes in hydrocephalus after hypoxia seems negligible. However, there was an increased vascular endothelial growth factor (VEGF) immune response in reactive astrocytes (Del Bigio et al., 2012). Increased expression of VEGF has also been reported in CSF of posthemorrhagic hydrocephalus in premature infants (Ballabh et al., 2007). Thus, VEGF-induced angiogenesis may be an alternative mechanism for hypoxic tissue protection. VEGF has been proposed as a treatment for hypoxia. However, in animal models, injections of VEGF have been shown to cause ventricular enlargement (Harrigan et al., 2002; Shim et al., 2013).

In the case of brain injury, astrocytes provide antioxidant protection, such as the secretion of neurotrophic factors, antioxidants, and so on (Cabezas et al., 2019). Astrocytes contain high concentrations of antioxidants such as glutathione(GSH), which can remove excess ROS (Dringen, 2000). There is also evidence that astrocytes release glutathione precursors, which neurons use for glutathione synthesis (Dringen et al., 1999). IL-1β may stimulate the production of GSH in astrocytes through a process dependent on NF-κB, thereby enhancing the antioxidant capacity of tissues (He et al., 2015). When GSH depletion occurs, astrocytes and neurons are affected, with the latter being greatly influenced (González-Reyes et al., 2017). This neurotoxicity reflects the antioxidant dependence of neurons on astrocytes.

**FIGURE 2:**
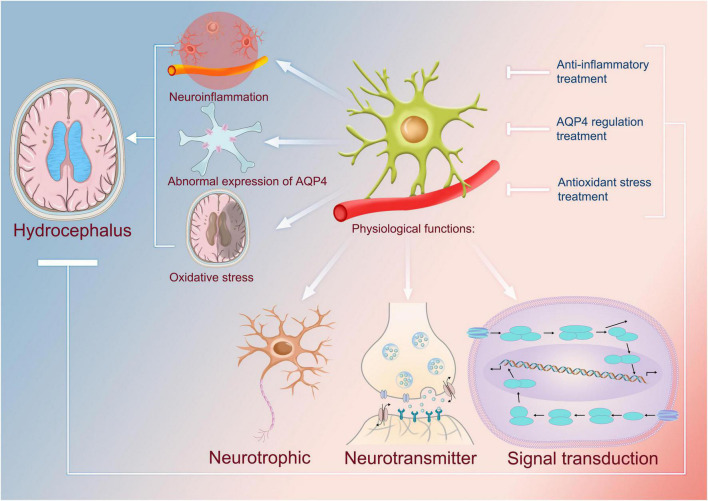
Physiological functions of astrocytes and their uses as targets in the treatment of hydrocephalus. The physiological functions of astrocytes include neurotrophic, neurotransmitter, and cellular signal transduction. It has been found that abnormal astrocytes are closely associated with the formation of hydrocephalus, and for some of the mechanisms identified so far, they can be involved in the development of neuroinflammation; therefore, anti-inflammatory therapies that use astrocytes as targets can be one of the means by which we treat hydrocephalus. Astrocytes are capable of causing abnormal overexpression of AQP4, which in turn leads to excessive accumulation of cerebrospinal fluid and causes hydrocephalus. Therefore, using astrocytes as a target to regulate the expression of AQP4 can also regulate the production of cerebrospinal fluid, which in turn can be used to treat hydrocephalus. In addition, astrocytes are also associated with oxidation, which contributes to hydrocephalus. Thus, it is safe to say that antioxidant therapy associated with them may be an effective treatment. AQP4, aquaporin 4.

In iNPH, Aβ deposition appears in the cerebral cortex (Tan et al., 2021). Recent studies on astrocytes have shown that astrocytes can also secrete Aβ (Frost and Li, 2017; Sanchez-Mico et al., 2021). Increases in pro-inflammatory cytokines seem to gradually lead to significant increases in post-translational levels of amyloid precursor protein and secreted Aβ (Zhao et al., 2011). This suggests that the persistent presence of inflammatory mediators may lead to dysfunction in astrocyte metabolism and production of Aβ, thereby aggravating oxidative stress (Figure 2).

## Therapeutic targeting of astrocytes for the treatment of hydrocephalus

At present, the mainstay of hydrocephalus treatment is surgery, and the research on non-surgical treatment has not achieved good results (Del Bigio and Di Curzio, 2016). Astrocytes can be involved in the pathological process of hydrocephalus in various ways, but there are still few studies targeting astrocytes. Current studies on astrocytes have mainly focused on the regulation of astrocyte-mediated neuroinflammation, abnormal expression of water channels, and oxidative stress.

Astrocyte-mediated neuroinflammation plays a role in the development of hydrocephalus, and some anti-inflammatory drugs seem to have a certain therapeutic effect. Minocycline is the second-generation tetracyclines. Minocycline is a highly lipophilic compound that can easily penetrate the BBB (Yong et al., 2004). Minocycline as a neuroprotective agent has been widely studied (Garrido-Mesa et al., 2013). Minocycline is reported to inhibit reactive gliosis and ventricular dilation in rat models of hydrocephalus (Xu et al., 2012a; Guo et al., 2015; Gu et al., 2019) and may provide additional benefits when used as a supplement for the ventricular shunt. However, considering that the studies on minocycline mainly focus on its inhibition of microglia activation and thus inhibit inflammation and other responses (Garrido-Mesa et al., 2013), the inhibitory effect of minocycline on reactive astrocytes in hydrocephalus might be caused by the regulation of expression of the pro-inflammatory factor of microglia.

Increasing AQP4 expression to accelerate CSF clearance appears to slow the development of hydrocephalus. Erythropoietin (EPO) treatment upregulated AQP4 expression and reduced ventricular dilation in kaolin-induced rat models of obstructive hydrocephalus (Rizwan Siddiqui et al., 2018; Suryaningtyas et al., 2019). EPO might decrease the expression of miR-130a and increase the expression of miR-668 (Rizwan Siddiqui et al., 2018). Upregulation of AQP4 may be a way to accelerate CSF clearance to treat hydrocephalus. However, maintaining AQP4 polarization in the endfeet of astrocytes may also be as therapeutic as simply increasing the abundance of AQP4.

The duality of astrocytes in oxidative stress makes them a good target for regulating the oxidative stress response. Edaravone is an excellent antioxidant that inhibits the production of free radicals, thereby preventing cell death caused by oxidative stress (Wang et al., 2011; Kikuchi et al., 2017). It is reported that treatment with edaravone for 14 days after hydrocephalus induction can reduce the activity of astrocytes on the corpus callosum and germinal matrix (Garcia et al., 2017). However, the dose of the drug used did not show antioxidant ability. There is also evidence that edaravone can inhibit the development of hydrocephalus by activating the Nrf2/HO-1 signaling pathway to protect ependymal cilia and neurons from oxidative stress damage (Zhang et al., 2018). Some natural extracts with antioxidant properties have conflicting therapeutic benefits (Catalão et al., 2014; Sampaio et al., 2019). Interestingly, the oral antioxidant mixture α-tocopherol, L-ascorbic acid, coenzyme Q10, reduced glutathione, and reduced lipoic acid showed no therapeutic benefits for juvenile rats with kaolin-induced hydrocephalus (Di Curzio et al., 2014). Further, there was no evidence in this study suggesting that the antioxidant treatment reduced the astrocyte response. This may be due to lower peak levels of oral therapy than after parenteral administration. In other studies, hydrocephalic young rats treated with hyperbaric oxygen therapy performed better on behavioral tests than untreated rats, although there was no significant effect on ventricular dilation (da Silva et al., 2018). Silva et al. suggested that hyperbaric oxygen therapy may promote functional recovery of the CNS by inhibiting the activation of astrocytes and forming an extensive fibrillar network, in addition to its own antioxidant stress effect (da Silva et al., 2018; Supplementary Table 1).

In addition to the known therapeutic measures mentioned above, based on the analytical elaboration of the role of astrocytes above, we can use this as a target to consider the choice of therapeutic measures in future studies. For example, developing drugs to act on astrocytes to clear neuroinflammation, targeting astrocyte AQP4 expression to address cerebrospinal fluid problems, and using certain antioxidants to clear free radicals may be possible. These could be the focus of research into new measures for the astrocyte-based treatment of hydrocephalus.

## Conclusion

Astrocytes play a key role in maintaining the normal function of the CNS. Astrocytes maintain the normal metabolism of the brain, regulate synaptic transmission and plasticity, and prevent neurons from producing toxic compounds. Recent studies have shown that the response of astrocytes in hydrocephalus is twofold and depends on its appearance period and specific signaling mechanisms. In the early stage of hydrocephalus, astrocytes can inhibit the spread of inflammation, show adaptive changes to the accumulation of CSF to enhance absorption, and release antioxidant substances to fight oxidative stress. However, as the disease progresses, reactive astrocytes release inflammatory mediators and promote oxidative stress. The abnormal expression of AQPs is also gradually harmful. Therefore, reactive astrocytes may be a potential target of therapeutic strategies for hydrocephalus. Despite that, the dual role of astrocytes complicates the study of their therapeutic effects. Stimulating glial activity in the early stages may yield good results. Notwithstanding, this activation in the late stages may worsen the disease. Therefore, grasping the right time window is the key to achieving its optimal effects.

Although we have searched for new astrocyte-based treatments for hydrocephalus and explored new approaches based on existing measures, we still do not know anything about the molecular mechanisms behind them, which may limit our thinking and thus require us to study them in depth.

In conclusion, we have discovered the important role of astrocytes in three aspects: participation in neuroinflammation, regulation of water molecule proteins, and antioxidation, which are also factors in the pathogenesis of hydrocephalus; thus, astrocytes can be used as targets for us to investigate new methods of drug treatment for hydrocephalus, and as a result, numerous highly effective drugs can be developed.

## Author contributions

YJY, CSW, and RC collected the related manuscript.YJY, RC, YCW, CWT, CSW, and JPL drafted and revised the manuscript. QHZ and GLX participated in the review design and helped draft and revise the manuscript. All authors have read and approved the final manuscript.
